# The relationship between knowledge of malaria transmission and malaria prevention and the risk of malaria infection in the coastal region of Batam City in Indonesia

**DOI:** 10.1186/1475-2875-9-S2-P50

**Published:** 2010-10-20

**Authors:** Dewi Susanna, Tris Eryando

**Affiliations:** 1 Department of Environmental Health, Faculty of Public Health, University of Indonesia, Depok Campus, Indonesia 16424; 2 Department Biostatistic and Health Informatics, Faculty of Public Health, University of Indonesia, Depok Campus, Indonesia 16424

## Backgound

Malaria is still become a serious health problem in Indonesia. The spread of malaria cases are depend on many factors, such as area characteristics or ecological factors. The objective of this study was to assess the relationship between the knowledge of malaria transmission and malaria prevention and the risk of malaria infection in the coastal region of Batam City in Indonesia.

## Methods

The study was conducted in coastal region the coastal region of Batam City in Indonesia, using crossectional design. This research had got the subject 170 in total included cases and non-cases. The case detection based on the microscopy examination. Then, the subjects were asked using a structured questionnaire consisted of 22 questions regarding the knowledge, perception, and practice everything about malaria. The obtained data was managed based on the median of the all answers and then categorized as good and bad knowledge. The collected data analyzed using χ^2^ test and spatial analyses using nearest neighbours by Thiessen polygons analysis [1].

## Results

There was no significant relationship (p > 0.05) between the variables of general knowledge, malaria transmission, malaria prevention with number of cases and non-cases. The knowledge on malaria transmission did not have relationship with malaria incidence (Table 1). From the polygon analysis seemed the distribution of malaria cases and no cases appeared evenly spread throughout the area of research.

**Table 1:** The Relationship between knowledge of malaria transmission and malaria prevention and the risk of malaria infection in the coastal region of Batam City in Indonesia

	Malaria				
				
Variable	Case	Non Case	Total	p value	OR (CI 95%)
General Knowledge					
Bad	15	65	**so;**		
Good	11	79	90	0,23	1,66 (0,66-4,19
Total	26	144	170		
Transmission					
Bad Knowledge	18	111	129		
Good Knowledge	8	33	41	0,389	0,67 (0,25-1,86)
Total	26	144	170		
Prevention					
Bad Knowledge	20	113	133		
Good Knowledge	6	31	37	0,860	0,93 (0,40-2,14)
Total	26	144	170		

OR, Odds ratio; CI, Confidence Interval 95 %

## Conclusion

There were not the differences between knowledge of malaria transmission and malaria prevention and the risk of malaria infection in the coastal region. The cases and non cases distribution seemed spread evenly.
